# New morphological information on, and species of placoderm fish *Africanaspis* (Arthrodira, Placodermi) from the Late Devonian of South Africa

**DOI:** 10.1371/journal.pone.0173169

**Published:** 2017-04-05

**Authors:** Robert W. Gess, Kate M. Trinajstic

**Affiliations:** 1 Geology Department, Rhodes University, Grahamstown, South Africa; 2 DST-NRF Centre of Excellence in Palaeosciences (CoE-Pal) University of the Witswatersrand 1 Jan Smuts Avenue Braamfontein Johannesburg, South Africa; 3 Albany Museum, Somerset St, Grahamstown, South Africa; 4 Department of Environment and Agriculture, Curtin University, Perth, Western Australia, Australia; Royal Belgian Institute of Natural Sciences, BELGIUM

## Abstract

Here we present a new species of placoderm fish, *Africanaspis edmountaini* sp. nov., and redescribe *Africanaspis doryssa* on the basis of new material collected from the type locality of *Africanaspis*. The new material includes the first head shields of *Africanaspis doryssa* in addition to soft anatomy for both taxa. Hitherto *Africanaspis* was entirely described from trunk armour and no record of body and fin outlines had been recorded. In addition the first record of embryonic and juvenile specimens of *Africanaspis doryssa* is presented and provides a growth series from presumed hatchlings to presumed adults. The presence of a greater number of juveniles compared to adults indicates that the Waterloo Farm fossil site in South Africa represents the first nursery site of arthrodire placoderms known from a cold water environment. The preservation of an ontogenetic series demonstrates that variation within the earlier known sample, initially considered to have resulted from ontogenetic change, instead indicates the presence of a second, less common species *Africanaspis edmountaini* sp. nov. There is some faunal overlap between the Waterloo Farm fossil site and faunas described from Strud in Belgium and Red Hill, Pennsylvania, in north America, supporting the concept of a more cosmopolitan vertebrate fauna in the Famennian than earlier in the Devonian.

## Introduction

**Fig 1:**
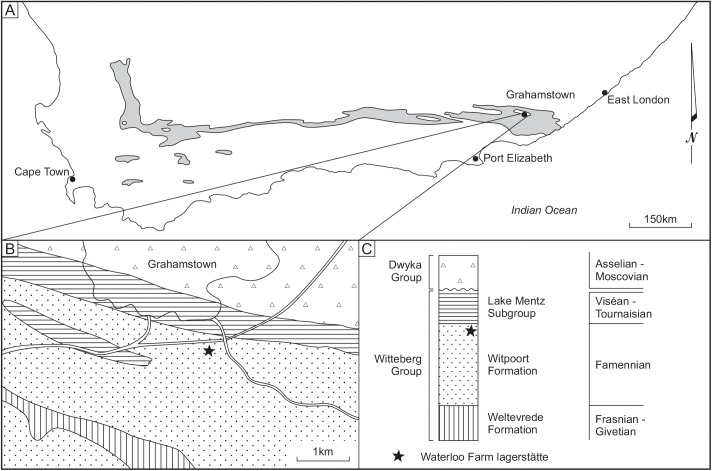
Locality map, geographic setting and stratigraphy of the fossil locality. A, location and distribution of the Witteberg Group in South Africa; B, location of the fossil site at Waterloo Farm lagerstätte (marked with a star); C, stratigraphic section showing the position of the Witpoort Formation within the Witteberg Goup.

Fossil fish remains were first recovered from the Late Famennian black shales of the Witpoort Formation at Waterloo Farm (Fig 1) following road excavations in 1985. An initial faunal report [1] identified, but did not describe, placoderms (*Bothriolepis*, groenlandaspidids and erroneously a phyllolepid and macropetalichyid [2]), gyracanthid acanthodians, chondrichthyans, “crossopterygians” and dipnoans[1]. A preliminary taxonomic catalogue more fully illustrated the rich and diverse fauna present [3] and descriptions of the Placodermi; *Bothriolepis africana*, *Groenlandaspis riniensis* and *Africanaspis doryssa* [2], and a chondrichthyan *Plesioselachus macracanthus* [4] followed. Further collecting and preparation of shales from the Waterloo Farm locality is revealing far greater diversity and description of new material is ongoing. Additional vertebrate taxa described from the assemblage have expanded the fauna to include another acanthodian *Diplacanthus acus* [5], a lamprey, *Priscomyzon riniensis* [6], a second chondrichthyan *Antarctilamna ultima* [7] and a coelacanth, *Serenichthys kowiensis* [8]. New material has also permitted a complete reevaluation of *Plesioselachus* [9]. The fauna represents Africa’s most complete Late Devonian aquatic community and currently is the only known high latitude estuarine paleoenvironment of Famennian age [10].

Subsequent field work has resulted in further placoderm material being recovered, including more complete and associated specimens, belonging to the genus *Africanaspis* [2]. New material includes; the first recognised head shields of *Africanaspis*, head and trunk shield plates from juveniles, including an apparent hatchling and impressions of soft tissue from the body, fins, tail and eyes. The soft tissue impressions provide the first non-skeletal morphological information on the post cranial body for any groenlandaspidid-like taxa. The new material indicates that there is more than one species of *Africanaspis* and the objectives of this paper are to provide a more complete description of *Africanaspis doryssa*, and to describe the second species of *Africanaspis*.

Within extant fishes, up to five developmental stages (embryonic, larval, juvenile, adult and senescence) may be present; however, the identification of these developmental stages within fossil taxa is problematic [11]. Within fossils, size is commonly used as an approximation of age with smaller fish of the same species considered to represent juveniles [12]. To date the free living larval stage (individuals having the egg yolk still attached) has only been reported for antiarchs (basal placoderms) based on the presence of an opening in the ventral trunk shield presumed to be the attachment area for the yolk sac [13]. For some arthrodire taxa the embryonic stage has been identified because intrauterine embryos have been recovered from within adult females [14–16]. Embryos of differing size have been recovered from *Incisoscutum ritchiei*, a coccosteomorph arthrodire, with the largest embryos approximately the size of the smallest individual (presumed free swimming) specimens [17]. Hatchlings, identified as such due to the lack of a yolk sac, from *Cowralepis mclachlani* also demonstrate the smallest size of the juveniles (6cm) of this taxon [18]. Here we describe several small specimens of *Africanaspis doryssa*, which are interpreted as representing juveniles, based on their size in comparison with adults, as well as embryonic specimens. Furthermore we demonstrate morphologies that characterise placoderms juveniles ([11] for a review). The paleoecology of the Waterloo Farm site, in particular the interpretation that this site represents a high latitude paleonursery, is considered.

### Geological setting

The Famennian Witpoort Formation (Witteberg Group, Cape Supergroup) occurs from near Fish River, south east of Grahamstown (Eastern Cape, South Africa) westward along the Cape fold belt to the Cedarberg in the Western Cape (Fig 1) [3]; [19]; [20]. It is the middle component of the Witteberg Group, which is the youngest of three main divisions of the tripartite Ordovician to Carboniferous Cape Supergroup (Fig 1B and 1C) [21]. The Witpoort Formation conformably overlies the Givetian-Frasnian Weltevrede Subgroup and in turn is overlain by the Tournaisian-Viséan formations of the Lake Mentz Subgroup [3]. The Witpoort Formation comprises predominately quartz arenites, wackes and subarkoses with minor interbeds of shale [21].

The depositional environment has been interpreted as representing a brackish coastal back-barrier lagoonal system with significant fresh water input, sheltered by a sandy barrier bar system [3]; [19]; [20].

## Materials and methods

### Field work

*Africanaspis* fossils were initially collected during excavations by RG, between 1993 and 1995 from a thick layer (up to 6 meters) of laminated black shale lenses near Grahamstown, in the Eastern Cape Province of South Africa. This layer was exposed in 1985 during construction of the N2 road bypass, in a cutting at Waterloo Farm (33°19'24.88"S, 26°32'12.70"E) (Fig 1). Following collapse of the cutting in 1999, 30 cubic metres of the shale was removed by hand during stabilisation of the road cutting. These were transported to a storage facility and have allowed for continued meticulous excavation to reveal the fossils. In 2006 a further collapse occurred allowing the removal of additional fossiliferous material to the storage facility, facilitated by the National Roads Agency. All *Africanaspis* material has been recovered from the ‘main fish lens’ (MFL), which has been the prime source of vertebrate material.

The field work was done in South Africa with permission obtained from the National Roads Agency of South Africa (the land owners), under a South African Heritage Resources Permit (80/06/11/005/61 issued under section 35(4) of the National heritage Act, Act no.25 of 1999 to collect at this site.

### Preservation of material

Fossils are preserved as near two-dimensional compressions, either as articulated, associated or isolated elements. The vertebrate bone was ultimately replaced by secondary metamorphic mica, which has variably been altered to kaolinite following uplift. Within the placoderms no remnants of the internal perichondral skeletal elements remain. Soft anatomy preservation occurs as white to grey/black impressions of variable clarity or as impressions.

Impressions of soft tissues and body outlines occurred when pulses of mud buried their remains rapidly in fine-grained anoxic lagoonal bottom sediments. Regular, possibly riverine, supply of fine sediment is reflected in a thick stack of sediment [21]. When deposition was more gradual or carcasses were larger they were variably reduced by decay and scavenging with the resistant elements of larger individuals often widely scattered prior to burial.

### Preparation and imaging

Black shale layers were split apart and where necessary matrix covering the dermal plates, or soft tissue impressions, was manually removed. Specimens, prepared post 1997, were photographed under various light conditions and angles (with a Nikon D80 camera and a Micronikor 60 mm lens), in order to best show the morphological features. Previously collected specimens were prepared in 1994 and 1996 by washing the kaolinite away with 70% alcohol solution, manually removing clay particles, sealing the surface with a solution of 25% glyptal cement and acetone, and then making latex peels [2]. This process was not repeated on the material prepared after 1997 because the flattened nature of the preservation resulted in the latex peels showing little morphological detail. In addition, the preparation technique removed most of the soft tissue impressions. Nonetheless these pre 1997 specimens, including the type material of *Africaspis doryssa*, and photographs thereof taken (by RG) prior to this latter preparation, were utilised in this study.

Following preparation all specimens were registered in the collections of The Albany Museum as AM 5246; AM 5247; AM5905; AM 4907; AM 5924; AM 5923; AM 7502; AM7503; AM 5943; AM 5921; AM 5920; AM5242; AM5922. Specimen repository is located within the Albany Museum Somerset St, Grahamstown, 6139, South Africa.

### Nomenclatural acts

The electronic edition of this article conforms to the requirements of the amended International Code of Zoological Nomenclature, and hence the new names contained herein are available under that Code from the electronic edition of this article. This published work and the nomenclatural acts it contains have been registered in ZooBank, the online registration system for the ICZN. The ZooBank LSIDs (Life Science Identifiers) can be resolved and the associated information viewed through any standard web browser by appending the LSID to the prefix "http://zoobank.org/". The LSID for this publication is: urn:lsid:zoobank.org:pub: 562DF258-104A-4B38-A731-BF151C1A0570. The electronic edition of this work was published in a journal with an ISSN, and has been archived and is available from the following digital repositories: PubMed Central, LOCKSS.

#### Anatomical Abbreviations

**ADL,** anterior dorsolateral plate; **ADL. un**, anterior dorsolateral plate underlap; **af,** anal fin; **AL,** anterior lateral plate; **AL ov.,** overlap area for the anterior lateral plate; **APi,** anterior pineal plate; **art.c,** articular condyle on the ADL; **AVL,** anterior ventrolateral plate; **b.i.,** body impression; **Ce,** central plate; **cf,** caudal fin; **csc,** central sensory canal; **df,** dorsal fin; **?g,** possible gut; **ioc,** infraorbital canal; **kd,** glenoid condyle; **lc,** main lateral line canal; **M,** marginal plate; **M ov,** overlap area for the marginal plate; **MD,** median dorsal plate; **mt**, mineralised tissue; **Nu,** nuchal plate; **Or,** orbit; **orb m**, orbital margin; **ot c,** otic capsule; **pd**, posterior denticles on the median dorsal plate; **PDL,** posterior dorsolateral plate; **PDL. un**, posterior dorsolateral plate underlap; **pf,** pectoral fin; **PL,** posterior lateral plate; **PL ov,** overlap for the posterior lateral plate; **PM,** post marginal plate; **PM ov,** overlap area for the post marginal plate; **pmc,** postmarginal sensory canal; **PN,** post nasal plate; **PNu,** paranuchal plate; **pp,** posterior pit line groove; **PPi,** posterior pineal plate; **PrO,** preorbital plate; **PtO,** postorbital plate; **PtO ov,** overlap area for the postorbital plate; **PVL,** posterior ventrolateral plate; **R,** rostral plate; **rdg,** ridge; **SG,** supragnathal **SO,** suborbital plate; **soc,** supraorbital sensory canal; **Sp,** spinal plate; **vbw**.

#### Institutional Abbreviation

**AM**, Albany Museum, Grahamstown.

## Results

### Systematic paleontology

Placodermi Woodward, 1891 [22]
Arthrodira Woodward, 1891 [23]
Suborder Phlyctaenioidei Miles, 1973 [24]
Infraorder Phlyctaenii Miles, 1973 [24]
Family Groenlandaspididae Obruchev, 1964 [25]
***Africanaspis*** Long, Anderson, Gess, Hiller, 1997 [2]

#### Type locality

Witpoort Formation (Witteberg Group, Cape Supergroup) from Waterloo Farm, south east of Grahamstown, Eastern Cape, South Africa (Fig 1).

#### Horizon & age

Late Famennian, Devonian

### Revised generic diagnosis

Short, high trunk covers the anterior body, posterior to thoracic armour the body tapers and there is a long whip-like tail. Anterior dorsolateral plate with relatively straight anterior margin. Step on anterior dorsolateral plates accommodates the anterior margin of the median dorsal plate. No overlap between left and right Anterior dorsolateral plates. Anterior and posterior dorsolateral plates with straight medial margins. Tall posterior dorsolateral plate, posterior margin concave in lateral view, distinct shoulder ventral to the posterior contact with the median dorsal plate. Median dorsal plate higher than long, pointed and slightly anteriorly posteriorly re-curved. Median dorsal anterior margin smooth, posterior margin ornamented with a serration-like series of modified denticles. The anterior lateral plate is large, sutured to the entire width of the lateral trunk armour, and laterally angled.

#### Remarks

The diagnosis of this genus has been emended from Long et al.[2] to comply with observations, based on new material, made herein.

**Fig 2:**
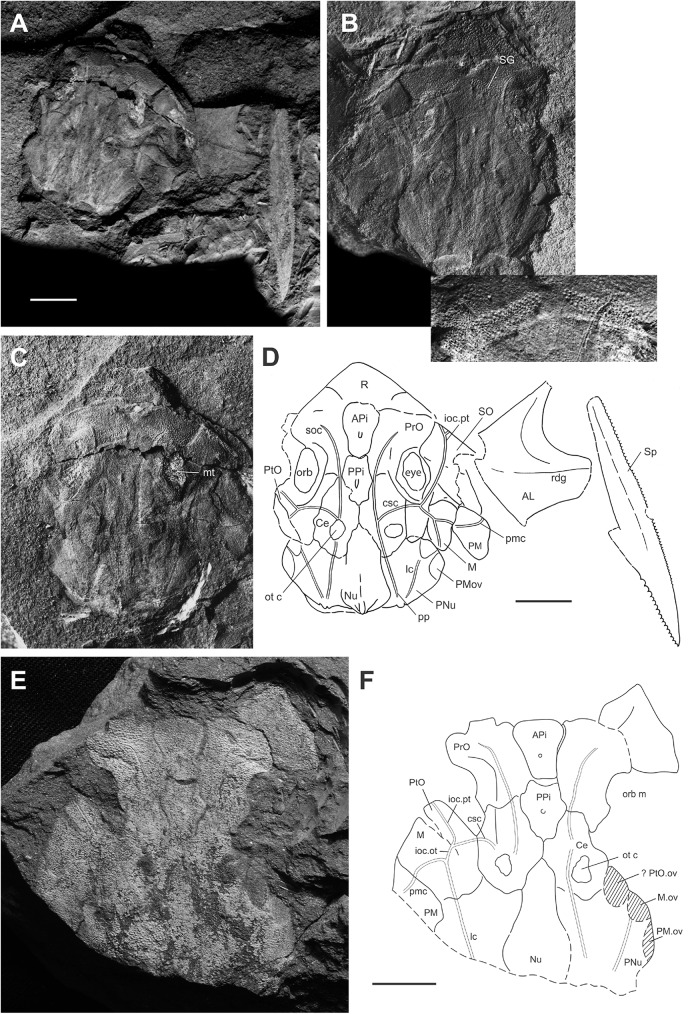
Skull roof and cheek bones of *Africanaspis doryssa*. A, internal dorsal view AM 4907a (part); B, dorsal view 4907b (counterpart) with insert showing detail of the supragnathal plates; C, external dorsal view AM 4907a; D, outline drawing based on 4907a and 4907b; E, external dorsal view AM 5943; F, outline drawing based on AM 5943. scale bars = 1 cm.

**Fig 3:**
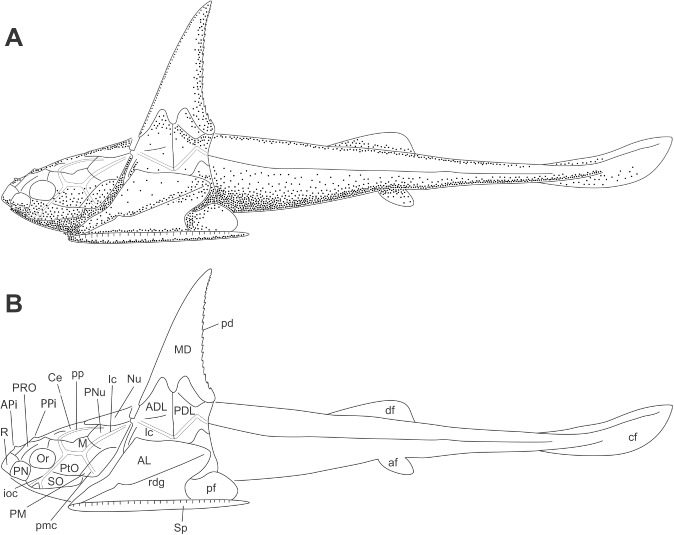
Reconstruction of *Africanaspis doryssa*. A. whole body reconstruction in lateral view based on described material, form of pectoral and caudal fins hypothetical, body partially based on that of *A*. *edmountaini* (below); B, outline drawing (not to scale).

**Fig 4:**
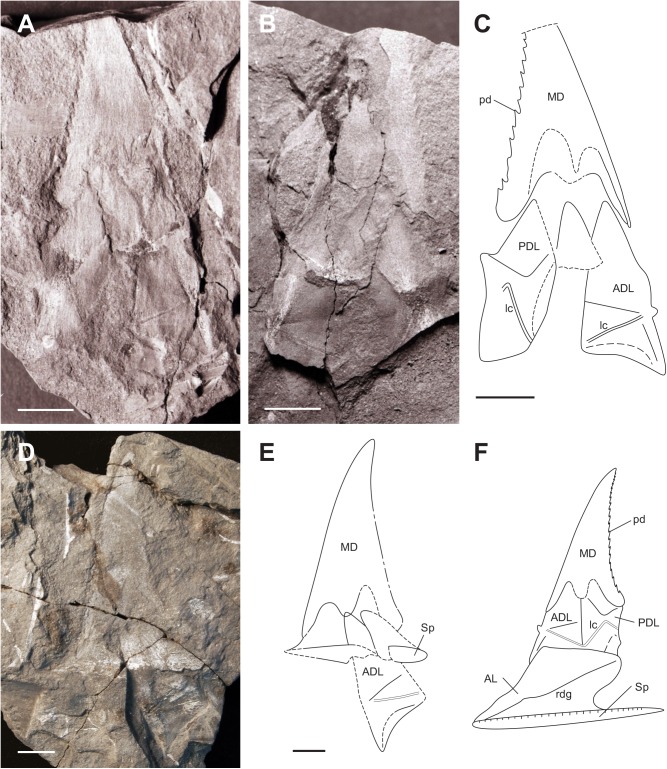
Trunk armour of *Africanaspis doryssa*. A, lateral view of the trunk armour AM 5246a part (holotype); B, lateral view of the trunk armour AM 5246b counterpart; C, outline drawing of the holotype based on AM 5246a &b; D, lateral view of the trunk armour of the paratype AM5247; E, outline drawing of the paratype based on AM5247; F, reconstruction of trunk armour based on AM 5246 and AM5247, with AL and spinal reconstructed from AM 4907 (Fig 2) and AM 5905 (Fig 5). scale bars = 1 cm.

**Fig 5:**
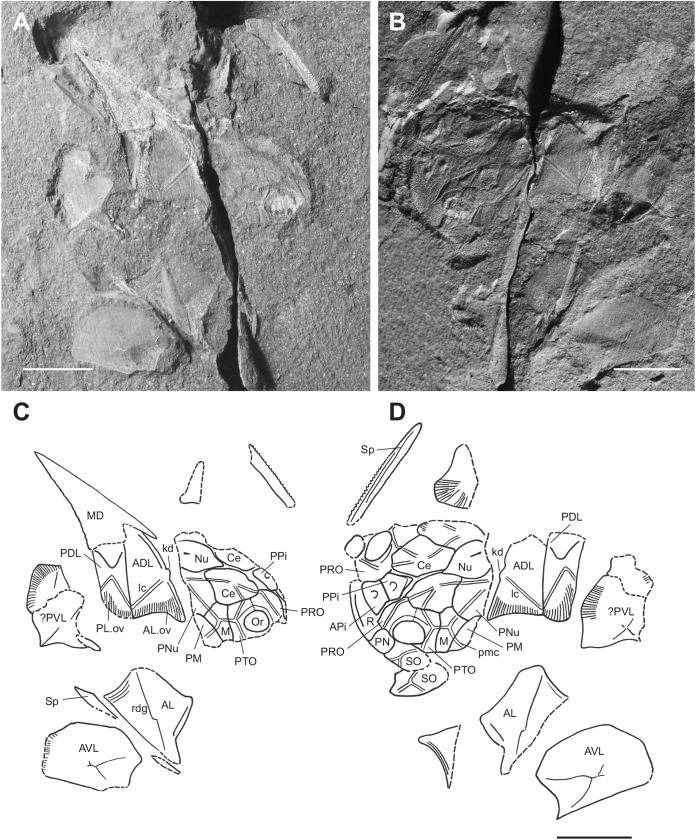
Juvenile specimen of *Africanaspis doryssa*. A, lateral view of head and trunk shield of AM 5905a (part); B, dorsal view of the head and trunk shield AM 5905b (counterpart); C, outline drawing based on AM 5905a; D, outline drawing based on AM 5905b scale bars = 1 cm.

**Fig 6:**
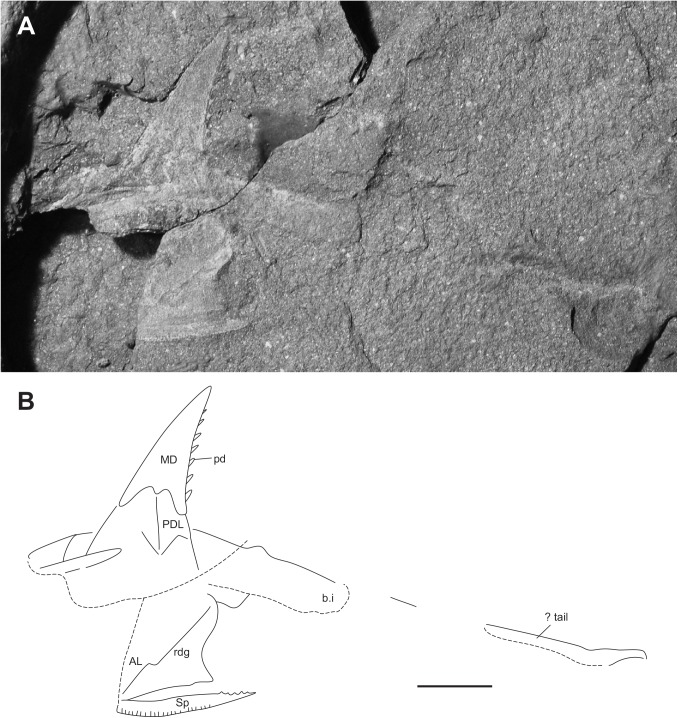
Trunk armour and body outline of *Africanaspis doryssa*. A, partial semi-articulated subadult, AM 5702; B, outline drawing of AM 5702 scale bar = 1cm.

**Fig 7:**
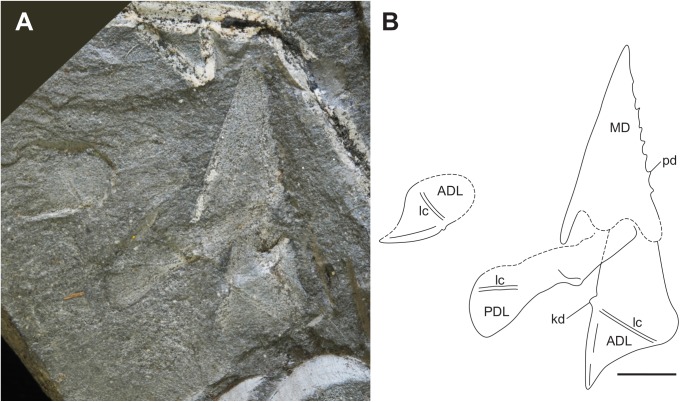
Trunk shield of a juvenile specimen of *Africanaspis doryssa*. A, disassociated trunk armour plates, AM 5923; B, outline drawing of AM 5923. scale bar = 1 cm.

**Fig 8:**
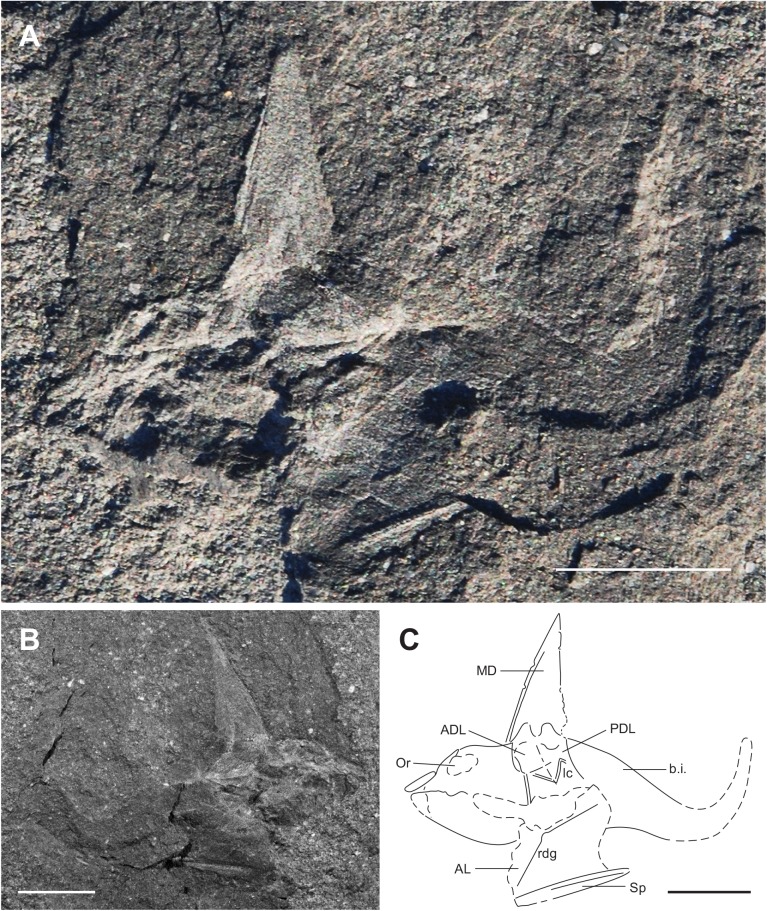
Neonatal specimen of *Africanaspis doryssa*. A, lateral view of the whole body AM 7503a part; B, lateral view of the head and trunk shield AM 7503 b; C, reconstruction of a neonatal specimen of *Africanaspis doryssa* based on AM 7503a & b. scale bar = 5mm.

***Africanaspis doryssa*** Long, Anderson, Gess, Hiller, 1997 [2] (Figs 2–8)

#### Synonymy

*Groenlandaspis* sp. Gess and Hiller, 1995 [3]

*Groenlandaspis riniensis* Long et al., 1997 [2]

#### Holotype

AM 5246, by original designation. Comprises incomplete median dorsal plate with slightly displace anterior dorsolateral and posterior dorsolateral plates (see redescription below).

#### Additional material

AM 5247, MD plate with displaced anterior dorsolateral plate; AM5905 complete partially dissociated juvenile; AM 4907 a & b complete skull roof with M, and PM plates, tooth plates, Sp and AL plates; AM 5924, MD plate; AM 5923, MD plate, PDL and ADL plates; AM 7502 largely articulated partially preserved, MD plate, ADL, PDL, AL, Sp, Nu plates and dorsal tail outline; AM7503 wholebodied hatchling. AM 5943; complete skull roof with cheek plates.

#### Remarks

The recent recovery of multiple arthrodire juveniles, including embryos, from Latvia, Canada and Australia [11, 12, 14, 16–18, 26] has enabled a suit of characters including; thin dermal plates with large overlap areas, ornament restricted to the middle of the plate, proportionally larger head and orbits, and deeply incised sensory lines to be identified as characteristic of embryonic and juvenile placoderms. The specimen AM4907 does not possess any of these juvenile characteristics, indicating that it represents a mature individual. Furthermore associated trunk plates match those of *A*. *doryssa*. Thus the specimen AM4907 is here identified as *A*. *doryssa*, based on the revised diagnosis below whereas previously it had been assigned as a juvenile *Groenlandaspis riniensis* [2]

AM 5943 is tentatively identified as *A*. *doryssa* based on the similarity of the head shield plates with the above referred material, however no trunk shield plates were recovered with this specimen.

### Revised species diagnosis

*Africanaspis* with skull roof longer than wide (*although both specimens are flattened so determining a precise L/B ratio calculation is impossible*). Head armour approximately twice the length of trunk armour in dorsal view *contra* [2]. Rostral plate with strong dorsoventral inflection and paired, large postnasal plates form the face. Two medial pineal plates, separated by paired central plates, from a long narrow nuchal plate with posterior medial ridge. Preorbital plates the same length as the paranuchal plates. Suborbital plate forms ventral cheek margin, postmarginal plate forms posterior cheek margin. Similar sized marginal and postorbital plates. High, narrow dorsal trunk shield comprising single median dorsal plate (L: H 1:>2 with height measured obliquely along the front edge of the MD plate [34]) with well separated, upwardly projecting and acutely pointed serrations on the caudal margin. Lateral trunk shield short and high. Median dorsal plate ornament comprises fine tuberculated ridges whereas trunk ornament comprises domed tubercules connected by raised ridges. ADL plate with anterior and posterior margins meeting at an acute angle dorsally, articular condyle one third of the distance dorsally along anterior margin, and step for overlap of MD plate one third of the distance between condyle and dorsal apex. Anterior ventrolateral plates are longer than posterior ventrolateral plates, which have a high pectoral lamina enclosing the pectoral fin.

#### Remarks

The diagnosis of this species has been emended from Long et al. [2] to comply with observations, based on new material, made herein. Knowledge of the ventral armour is derived solely from the juvenile specimen (AM5905) and comprises the anterior ventrolateral plate and posterior ventrolateral plate.

### Description of adult specimens

#### Dermal bones of the skull and cheek (Figs 2 and 3)

In dorsal view the anterior margin of the skull roof is rounded (Fig 2A–2F). There is a strong dorsoventral inflexion along the anterior portion of the preorbital plates (Figs 2A and 2C–2F and 3A and 3B) and rostral plate (Fig 2B) with these, along with postnasal plates forming the face (Fig 3A and 3B). The exact shape of the postnasal plate, or if the postnasal and rostral plates are fused in the adult could not be determined due to poor preservation (Fig 2C and 2D). The skull roof, between the orbits is flat but becomes slightly arched about the rostrocaudal axis passing through the nuchal and paranuchal plates (Fig 2A–2F). The overlapping nature of the plate sutures indicates that the plates of the adult skull roof fit tightly together. Where preserved, the ornamentation comprises small sharp tubercles that are more concentrated on the external margins of the dermal plates (Fig 2A–2F).

The rostral plate is partially preserved in AM 4907b (Fig 2B), its ventrally directed triangular anterior margin being deflected up into the same plane as the skull roof during preservation. It posteriorly overlaps a large and anteriorly broad anterior pineal plate, which in turn underlaps a smaller and narrower posterior pineal plate (Fig 2A–2D). Similar shaped anterior and posterior pineal plates are also preserved in AM 5943 (Fig 2E and 2F). There is no pineal opening; however, in the centre of each pineal plate is a raised, dome like area of dermal bone (Fig 2B–2F) and on the visceral surface there is a small pit. These pineal plates separate paired preorbital plates (Fig 2), which form the dorsal and anterior portion of the orbit (Fig 3) and there is a concentration of tubercles around the orbital margins (Fig 2E). The anterior margin of the preorbital plate is broader than the posterior margin, and there is a posterior lobe on the posterolateral margin of the plate, where it extends its contribution to the margin of the orbit (Fig 2E and 2F). The paired central plates meet mesially, behind the posterior pineal plate (Fig 2B, 2D and 2F). A narrow, long median nuchal plate separates large, irregularly shaped paired paranuchal plates and these plates form the posterior margin of the skull roof (Fig 2A–2F). The anterior margin of the nuchal plate is acutely pointed. The posterior margin is not preserved in AM 5943 (Fig 2E and 2F), but in AM 4907 it is raised to form a medial ridge (Fig 2B–2D).

The postorbital plates predominately occupy a lateral position on the cheek (Figs 2C and 2D and 3). They have a strong dorsoventral inflection allowing the plate to curve around the posterior and posteroventral margins of the orbit (Fig 3A and 3B). The contact margin between the postorbital and marginal plates cannot be determined from either adult specimen, however both plates appear to overlap the paranuchal plate in the adult, fitting into a pair of embayed overlap areas. The postmarginal plate forms the posterior of the cheek and although the margins are not clear in AM 5943 (Fig 2E and 2F) it is posteriorly complete in AM 4907 (Fig 2C and 2D). It is higher than long and forms part of the posterior lateral margin of the skull (Fig 3). The large ovoid suborbital plate is best preserved in AM4907 (Fig 2C and 2D), although it is slightly displaced and it forms the ventral margin of the cheek and the anteroventral border of the orbit (Fig 3).

The path of the supraorbital sensory line canals extends from the anterior of the preorbital plates, onto the central plates (Fig 2B–2F). There is a small gap in the centre of the plate where the sensory canal is not apparent, before it reappears on the posterior portion of the central plate and terminates on the posterior of the paranuchal plate, near the main lateral line sensory canal. This pathway varies between the left and right sides of the specimen. The otic and postorbital region of the infraorbital sensory line canal extends from the suborbital plate to the middle of the cheek where they merge to join the main lateral line canal (Fig 2B–2F).

#### Dermal bones of the trunkshield (Figs 3 and 4)

A larger number of lateral trunk shield plates are preserved compared to the head shield plates with the median dorsal plate being the most commonly preserved of the trunk shield plates. The dorsolateral trunk armour is relatively short compared with *Groenlandaspis*, and in cross section it is bell-shaped. The single median dorsal plate is high with a narrow, bilobed base and in lateral view is triangular in shape (Fig 4A–4F). The anterior margin is straight to slightly convex and the caudal margin is fused with a series of well spaced, upwardly directed serrations (which decrease in size towards the apex) projecting from the caudal margin (Fig 4A and 4C). The plate ornament comprises low tubercles, which join together to form feint dorsoventrally oriented ridges (Fig 4A). There is a thin, ornamented anterior lamina that extends onto the anterior margin of the anterior dorsolateral plates somewhat above the articular condyle (Fig 4F). The suture, where the anterior and posterior dorsolateral plates underlap the median dorsal plate, is “m” shaped (Fig 4). The bone of the overlap area on the median dorsal plate is thinner than then rest of the plate and the outline of the high underlap areas of the anterior dorsolateral and posterior dorsolateral plates can be determined. The anterior dorsolateral plate is high and narrow, with a ventral margin which is concave - the curve fitting the dorsal margin of the anterior lateral plate (Fig 4). The anterior and posterior margins are straight and the posterior margin contacts with the anterior margin of the posterior dorsolateral plate (Figs 3 and 4A–4C and 4F). The articular condyle projects slightly upwards approximately two thirds down the anterior margin and there is a ridge running in a rostrocaudal direction from the condyle (Fig 4A, 4C and 4F). Approximately one third of the distance dorsally from the condyle, a step in the profile accommodates the overlapping anterior of the median dorsal plate (Figs 3 and 4). The lateral line of the main sensory line canal extends from the articular condyle obliquely downwards to near the contact with the anterior lateral plate (Figs 3 and 4A and 4D–4F). It continues dorsally at a slightly steeper angle onto the posterior dorsolateral plate on which it forms an inverted “V’ extending approximately halfway up the plate (Figs 3 and 4A and 4C). The posterior dorsolateral plate is approximately the same height and width as the anterior dorsolateral plate. There is a small posterodorsal process that locks behind the median dorsal plate (Fig 4A and 4C). The underlap area, where the posterior lateral plate overlaps, is represented by an area of lesser mineralisation of the posterior dorsolateral plate, curving around the caudal margin. It is more clearly represented in AM5905 (Fig 5). The posterior lateral plate itself has not been recovered. The anterior lateral plate is longer than high and the straight ventral margin is longer than the convexly curved dorsal margin (Figs 2D, 3 and 4F). The anterior margin is angled dorsocaudally at approximately 45° and the caudal margin is embayed to form an opening for the pectoral fin (Fig 3). There is a stepped ridge extending from the anteroventral corner diagonally upwards to the posterodorsal corner of the plate. The spinal plate is straight and extends caudally beyond the trunk armour, being 33% longer than the anterior lateral plate (Figs 2A and 2D, 3 and 4F). It tapers to a point caudally and exhibits small medially directed tooth-like denticles posterior to the anterior lateral plate. There is an ornament of well defined rounded tubercles, which form a pronounced closely spaced row along the outer lateral margin

#### Endocranial material and plates

Paired anterior supragnathal plates are preserved in life position, visible through the dermal surface of the preorbital plates in AM4907 (Fig 2B). The surface of the supragnathal plate is covered with denticles, radiating from the centre of ossification, which is located in the middle of the plate.

Within the orbit a mineralized white film covers the area that the eyeball would have occupied in life (Fig 2A).

The otic capsules show 3D preservation as black orbs visible through the posterior lateral portion of the central plates. No internal structure is discernible. The capsules appear to bulge through the dermal plates (Fig 2A) and so it cannot be determined if there is an otic ridge present on the dermal surface.

### Description of juvenile specimens

#### Dermal bones of the skull and cheek

The best preserved juvenile specimen is AM 5905 (Fig 5), however other informative juvenile specimens include AM 7502 (Fig 6) and AM5923 (Fig 7). The rostral plate forms the anterior margin of the head shield and is crescent shaped in dorsal view (Fig 5B and 5D). The pineal plates have not fully separated and a furrow runs between the anterior and posterior portion (Fig 5B and 5D). Both portions have a large medial boss. The anterior pineal plate is similar in shape to that of the adult (Fig 2D and 2F) though it is less elongate. The posterior pineal plate is posteriorly triangular, with an acute posterior margin (Fig 5B and 5D) whereas in the adult this plate is posteriorly broader and more rectangular (Fig 2D and 2F). The boss of both pineal plates is high and more prominent than those of the adult. The preorbital plates are shorter and narrower than in adults and do not extend to anteriorly contact the suborbital (Fig 5). The orbits are large. The supra orbital sensory canal is deeply etched into the dermal bone and extends across the central plates and onto the paranuchal plate (Fig 5B and 5D). The central plates are aligned mesially, however there is a gap between them (Fig 5B and 5D), possibly due to post mortem displacement and the anterior and lateral lobes are not as developed as in the adults (Fig 2). The central plates at this stage of development are the largest plates of the dermal skull roof (Fig 5). The nuchal plate is shorter than in adults, having an acute anterior margin and broad posterior margin (Fig 5). The paranuchal plates are large and the dermal neck joint well developed (Fig 5B and 5D). Thin overlap margins have formed between the plates of the skull roof, and define the plate boundaries. However, there are gaps between the ornamented surfaces present, which may indicate that the plates do not overlap at this developmental stage or that the ornament is yet to be fully developed.

The anterior margin of the postorbital plate forms the posterior margin of the eye, and the plate is limited to the cheek area of the head (Fig 5C and 5D). The margin between the postorbital and preorbital plates cannot be clearly distinguished. In the juvenile skull (AM 5905) the postorbital does not appear to contact the paranuchal plate (Fig 5) in contrast to the adult (cf Fig 4). The posteriorly incomplete suborbital plate is broad and forms the anteroventral margin of the orbit (Fig 5B and 5D). The postmarginal plate is anteriorly complete in AM5905 (Fig 5) whereas in the adult the posterior portion is preserved.

#### Dermal bones of the trunk shield

The median dorsal plate is high with a L:H ratio of 1:2, (Figs 5–7) which is consistent with the range recorded for the adult (and subadult) specimens (cf. Fig 4A and 4C). There is an anterior process on the median dorsal plate for anterior overlap of the anterior dorsolateral plate the extent of which is not clear as the specimens are partially dissociated (Figs 5–7). The dorsal margins of both the anterior and posterior dorsolateral plates are obscured by the median dorsal plate but the rest of the anterior median dorsal plate appears similar in morphology to that of the adult, being narrow and higher than long. The glenoid condyle is represented by a small bump on the anterior margin of the anterior dorsolateral plate (Fig 5A–5C). The plate overlap areas along the plate margins appear relatively larger than the overlap areas of the adult.

The anterior lateral plate is longer than high with a height to length ration of 0.66 (Fig 5). There is a distinct inflexion on the anterior margin, and dorsal to this inflexion the margin is relatively straight. Likewise the posterior margin of the plate is relatively straight in comparison to the adult plate where there is a deep concave curvature to allow for the fin insertion. This is consistent with considerable variation in plate shape described between other adult and juvenile arthrodires [27]; [28]; [18], and antiarchs [29–31]. There is a strong stepped diagonal linear ridge developed on the anterior lateral plate. The spinal plate is straight with small serrations occurring along the mesial margin and is 25% longer than the anterior lateral plate (Fig 5B–5D)

The anterior ventrolateral plate is longer than broad, with a straight margin where the spinal contacts and a high post -pectoral lamina (Fig 5). The anterior median ventral plate has not been recovered but there is a small curved indentation on the mesial margin for the anterior ventrolateral plate, suggesting presence of a triangular anterior median ventral plate. On the posterior margin of the plate there is a large overlap area for the posterior ventrolateral plate.

#### Body outline

The dorsal margin of the body is preserved in situ in AM 7502 and tissue impression extends from under the median dorsal plate laterally to the flank (Fig 6). The dorsal body impression is approximately five times the length of the dorsal trunk armour and the tail exhibits an upturned distal extremity, but the caudal fin is not preserved. The ventral body outline is not preserved possibly due, in part, to the thinner muscle wall and in part to rupture from the build up and release of gases of decomposition [32, 33]. This type of rupture has resulted in lack of preservation of part of the ventral body wall in *Austropyctodus* from the Gogo Formation, [16, 34].

### Description of neonate specimen (AM7503)

AM7503 (Fig 8) is a small wholebodied specimen identified as *A*. *doryssa* on the basis of its distinctive median dorsal plate, which entirely conforms to those diagnostic of this species (Fig 8). The interpretation of the specimen being a neonate is based on comparison with other arthrodire embryos [14, 16, 17]. Due to its size and the nature of its preservation it has not been possible to entirely prepare out its tail, and most plate boundaries are not well defined. It does provide an important indication of probable neonatal size, suggesting that the largest specimens collected are adults. The dorsal trunk armour is less than 4 mm in length and the median dorsal plate is approximately 9 mm high. Its body proportions are remarkably similar to those of adults with the exception of a large orbit. A sizeable mass beneath the head shield may include partially resorbed yolk material, indicating this individual would be transitioning from the larval to neonatal stage; however, this cannot be unambiguously ascertained. Notably the proportions of the median dorsal plate of all growth stages of *A*. *doryssa* are very consistent.

### Reconstruction and comparison of *Africanaspis doryssa*

#### Remarks

Although the use of juvenile specimens is not ideal in a composite reconstruction, where aspects of morphology are solely, or better understood in juvenile specimens, these have been incorporated.

### Head shield reconstruction (Figs 2 and 3)

The head reconstruction of *A*. *doryssa* (Fig 3) is based on the three head shields (Fig 2 showing the two adult headshields and Fig 5 the juvenile), the dorsal and dorsolateral trunk armour of AM5246 (Fig 5A–5C; the holotype) and AM5247 (Fig 4D–4E), the lateral trunk armour of AM 7502 (Fig 6) and AM 5923 (Fig 7) and the dorsal body outline of AM 7502 (Fig 6), in consultation with all known specimens. The size of the dermal head shield plates has been scaled to that of AM 4907, as this specimen was least distorted and had associated trunk shield plates. The size of the reconstructed trunk shield has been scaled to conform to the size of the anterior lateral and spinal plates preserved within AM 4907.

The head shield of *A*. *doryssa* is large, approximately twice as long as the trunk shield in dorsal view. In *Turrisaspis elektor* and *Tiaraspis subtilis* the head shield is 1.5 times longer than the trunk shield [35] whereas in *Groenlandaspis* the trunk shield is proportionally longer than the headshield. The arrangement of the dermal bones of the skull roof conforms to those in *Turrisaspis*, and *Tiaraspis*. Both *Turrisaspis* and *A*. *doryssa* have a V-shaped posterior skull margin, which is raised towards the median dorsal plate, although this is more pronounced in *Turrisaspis*, whereas *Tiaraspis* is reconstructed as having a straight posterior skull margin [35] as is characteristic of the groenlandaspidids.

There are two separated pineal plates in adults of *A*. *doryssa*. In juveniles, there is a deep furrow running between the pineal plates, though the plates are not clearly separated. The separation of the plates may not occur until later ontogeny. The presence of two pineal plates is reported for *Turrisaspis elektor* [36] and, although only one pineal plate is known from *Tiaraspis subtilis* there is a gap between the posterior margin of the rostral plate and the pineal plate [35] which indicates that two pineal plates may have also been present [36]. In *Groenlandaspis antarcticus* only one pineal plate, fitting tightly behind the rostral plate, is reported [37]. The possibly undifferentiated pineal plates in the juvenile of *Africanaspis*, and the presence of a dermal boss and visceral pit on both pineal plates in the adult indicate that the posterior plate is correctly identified as a pineal plate. Daeschler et al. [36] report variation in the shape and size of the pineal plates in adults of *Turrisaspis;* and similar variation is seen in adult skulls assigned to *A*. *doryssa*. The small more triangular shaped posterior pineal plate present in the juvenile (AM5905) (Fig 5) is considered an early ontogenetic feature because there are gaps around the lateral and posterior plate margins to allow for plate growth. Differences in the timing of ossification for the dermal plates, and gaps between plates which, allow for allometric growth has been reported for antiarchs [11, 31] and arthrodires [12, 14, 16–18].

The central plates of AM 4907 have been used in the reconstruction. The central plates in adult specimens occupy a similar proportion of the skull roof as in *Tiaraspis* and *Turrisaspis*. However, in the juvenile these are the largest plates of the skull roof, 1.5 times larger than the preorbital plates and approximately the same length as the paranuchal plates. Variation in the length of the posterior lobe on the central plate is seen between the adult specimens of *A*. *doryssa*. This variation is also present in *Tiaraspis subtilis* with well defined posterior lobes present in two specimens (Go 807-1, Go 807-3) but absent in Go 807-2 (fig 1 A-C; Gross, 1984 [38]) and in *Turrisaspis elektor* where the left posterior lobe is longer than the right (fig 7 A-B; [36]). Variation in the morphology of the central plate lobes has also been noted within the coccosteid arthrodires [27, 39].

The juvenile (AM5905) nuchal plate is shorter and broader than in adults; a morphology also present in the eubrachythoracid arthrodire *Compagopiscis croucheri* [12, 28]. The narrow nuchal plate present in AM 4907 (Fig 2D) is comparable to AM 5943 (Fig 2E) and so is here interpreted to represents the adult morphology of *A*. *doryssa*. The presence of a well defined median ridge and the lack of a median boss on the nuchal plate is similar to *T*. *elektor* [36] and *Tiaraspis* [35] although *Tiaraspis* differs from the other genera in that the nuchal plate is club-shaped and the caudal region of the plate narrows.

The preorbital plates extend onto the face and as in *Tiaraspis* incorporate the postnasal plates into their anterior margin. The preorbital plates participate in the orbital margins. The orbits are intermediate in size, being smaller than in *Tiaraspis* but larger than *Turrisaspis*. In *Tiaraspis* sclerotic rings form the orbital margin, but these have not been reported in *Turrisaspis* and are not preserved in specimens of *Africanaspis*. The orbits of the juvenile of *A*. *doryssa* are larger than those of the adult as is common amongst vertebrates [11, 12].

The cheek in *A*. *doryssa* is reconstructed as comprising the postorbital, marginal postmarginal and suborbital plates. The postorbital and marginal plates are larger than in *Tiaraspis* but as in *Tiaraspis* the marginal plate separates the postmarginal plate from the postorbital plate. In *Turrisaspis* the postorbital and postmarginal plates suture under the marginal plate, and form the ventral margin of the cheek and this feature is considered unique for the species [36]. Schultze [35] reconstructed *Tiaraspis subtilis* with both suborbital and submarginal plates, although it is noted that these were not recovered and that the large orbits leave little space for them. Indeed the space filled by the submarginal in Schultze’s [35] reconstruction is, in our opinion, more likely to have been filled by a tapering posterior extension of the suborbital. A suborbital plate is not described or reconstructed for *Turrisaspis* (fig 6C; [36]) although in the reconstruction presented the ventral margin of the orbit is open suggesting that a suborbital plate was present. Indeed in the photo of ANSP 20961 (fig 6A [36]) a bony plate is situated below the orbit, completing the orbital margin. In AM5905, a juvenile specimen of *A*. *doryssa*, a left suborbital plate is preserved close to life position and forms the anteroventral margin of the orbit. It would appear that the arrangement of the cheek plates in *A*. *doryssa* is intermediate between that reconstructed for *Tiaraspis* [35] and *Turrisaspis* [36].

The path of the sensory line canals in *A*. *doryssa* is similar to *Turrisaspis*, which is the only other taxon reported where the path of the supraorbital sensory line canal disappears across the central plates and then resumes on the paranuchal plate, having presumably passed beneath the outer surface of the plate. In *Turrisaspis* this character was also noted to be variable [36]. Significant variation in the path of the sensory line canals has been noted within the coccosteomorphs [27, 39].

Trunk shield reconstruction (Figs 3 and 4F)

The original reconstruction of the dorsal and lateral trunk armour of *A*. *doryssa* [2] is here considered to represent a composite of the two species *A*. *doryssa* and *A*. *edmountaini* sp. nov. (see below).

The tall median dorsal plate of *A*. *doryssa* is similar in proportions to those of *Turrisaspis* [36] *Tiaraspis* [38] and *Groenlandaspis thorezi* [40], all of which also separate the ADL plates. More typical of *Groenlandaspis*, the median dorsal plate of *Groenlandaspis antarcticus* forms a low cap uniting the left and right dorsolateral plates [37]. Separation of the ADL plates by the median dorsal plate differentiates these taxa from *Mithakaspis*, another high median dorsal plate bearing arthrodire. In this taxon the ADL plates overlap anterior to the median dorsal plate. In addition *Mithakaspis* uniquely has ADL and PDL plates that overlap the median dorsal plate, *contra* the normal placoderm condition [41].

The ornament on the median dorsal plate of *A*. *doryssa* comprises vertical striations whereas *Turrisaspis* [36] and *Tiaraspis* [38] have both horizontal wavy rows of ornament in addition to linear striations. Long [2] reconstructed *A*. *doryssa* to have an ornament of both horizontal wavy rows and linear striations (fig 13 [2]), whereas we have found no evidence for the presence of horizontal wavy rows.

The ornamented anterior process on the median dorsal plate that extends onto the anterior margin of anterior dorsolateral plate terminates higher above the articular condyle than illustrated by Long et al. [2] and both the anterior dorsolateral and posterior dorsolateral plates are higher and narrower than previously reconstructed [2]. *Turrisaspis*, *Tiaraspis* and *A*. *doryssa* therefore share the character of high and narrow dorsolateral plates of the trunk shield. These are remarkably distinct from the low antero-posteriorly broad dorsolateral plates typical of *Groenlandaspis* (eg.[37]), or the even more antero-posteriorly elongate form of the posterior dorsolateral plate of *Boomeraspis* [42]. Compared with *Tiaraspis* and *Turrisaspis*, *Africanaspis*, has the most vertical median dorsal plate, which is reflected in the form of its anterior dorsolateral plate. In *Africanaspis* the anterior margin of the anterior dorsolateral plate rises towards its apex in a straight line interrupted only by a slight step to accommodate the overlapping anterior ‘leg’ of the median dorsal plate. In *Turrisaspis*, by contrast, the median dorsal plate is strongly posteriorly inclined. In concord with this the anterior face of the anterior dorsolateral plate of *Turrisaspis* is strongly dorsoposteriorly deflected at a point midway between the articular condyle and the overlap area with the median dorsal plate. Similarly, whereas the overlap area between the anterior dorsolateral and posterior dorsolateral plates is vertical in *Africanaspis* it is dorsoposteriorly bowed in *Turrisaspis*. Although the anterior dorsolateral plate of *Groenlandaspis thorezi* is not well preserved it appears to have a dorsoposterior deflection of the anterior margin similar to that of *Turrisaspis* (fig 4A, [40]). The anterior margin of the anterior dorsolateral plate of *Tiaraspis* is apparently also dorsoposteriorly deflected, however in this third state the deflection is at the point of overlap with the median dorsal plate (fig 2,[43]). In *A*. *doryssa*, as in *Turrisaspis* and *Tiaraspis* [36, 38] a raised crest extends from the condyle of the anterior dorsolateral plate towards the caudal margin, being most clearly visible in AM 5247. This is also apparent in some species of *Groenlandaspis* such as *G*. *riniensis* [2]. The acute inflexion of the main sensory line canal on the posterior dorsolateral plate is similar to that seen in *Turrisaspis* [36] and the genus *Groenlandaspis* [37] whereas in *Tiaraspis* the inflection is apparently less acute, though present [38]. A difference between adult and juvenile specimens of *A*. *doryssa* is in the shape of the anterior margin of the anterior lateral plate, above the point of inflection, which is straighter in juveniles than in adults. It has been suggested that shape change between juveniles and adults may be associated with a change in the angle of the head shield relative to the trunk shield during growth in *Turrisaspis* [36]. This hypothesis is supported by *A*. *doryssa* adults having a prominent articular condyle, which connects the head and trunk shields, whereas in juveniles the articular condyle is small and does not protrude from the anterior dorsolateral plate suggesting that the head and trunk shields did not articulate in the same manner in juveniles and adults. The articular condyle in ptyctodont and arthrodire embryos is also less developed than in adults [14, 44].

### *Africanaspis edmountaini* sp. *nov*.

#### Etymology

For Edgar Donald Mountain 2^nd^ professor of geology at Rhodes University and childhood geological mentor of RG

#### Synonymy

1995 *“g*roenlandaspidid” Gess and Hiller [3] p 285, fig 52 E

1997 *Africanaspis doryssa* Long et al. [2] p. 262, fig 12 B

#### Holotype

**Fig 9:**
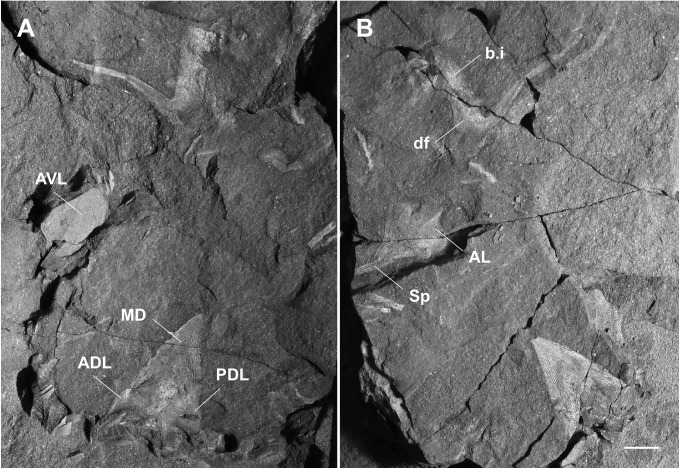
Holotype of *Africanaspis edmountaini sp*. *nov*. A, tail and disassociated trunk plate, AM 5921a; B, tail and trunk plates AM5921b. scale bar = 1cm.

**Fig 10:**
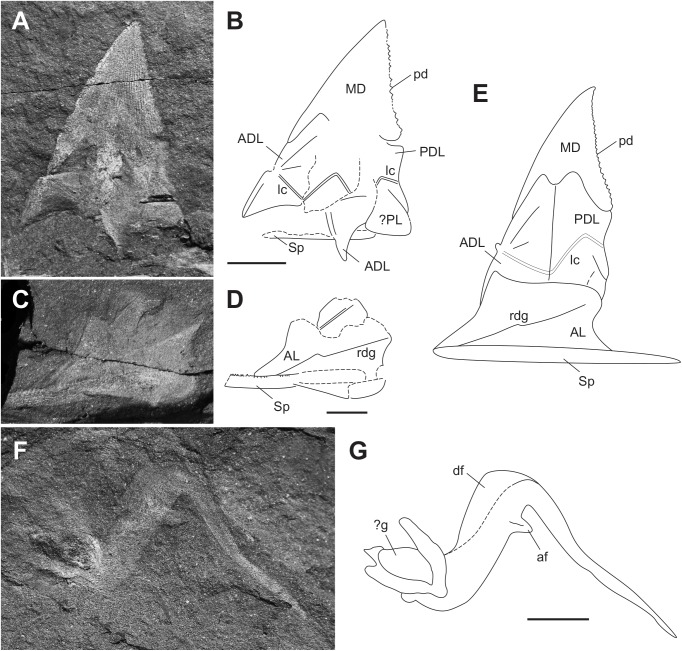
Details of the holotype of *Africanaspis edmountaini* sp. nov, A. Upper trunk armour plates in lateral view AM 5921a, B outline drawing of AM 5921 C, lower trunk armour plates in lateral view AM 5921b; D outline drawing of AM5921b; E, reconstruction of the lateral trunk armour based on AM 5921 a &b. F, Posterior body and tail in ventro-lateral view AM 5921; G, trunk reconstruction based on AM 5921 and AM 5920. scale bars = 1cm.

AM 5921 a & b (Figs 9 and 10) complete median dorsal plate, with slightly displaced anterior dorsolateral and posterior dorsolateral plates, dissociated anterior lateral plate, partially exposed spinal plate and partial anterior ventrolateral plate. Associated with these plates is the impression of the tail and single medial anal and dorsal fins.

#### Paratype

**Fig 11:**
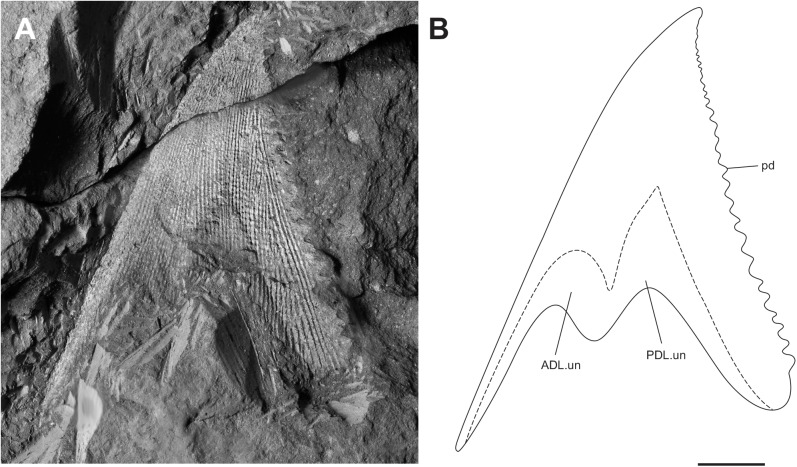
Median dorsal plate *Africanaspis edmountaini sp*. *nov*. A, the largest isolated median dorsal plate recovered AM 5920; B, Reconstruction of the median dorsal plate based on of AM 2920a and AM 5920b inverted.

AM 5920 a & b (Fig 11) complete median dorsal plate in part and counterpart.

#### Type locality and horizon

Witpoort Formation (Witteberg Group, Cape Supergroup), Waterloo Farm, Grahamstown, South Africa. Late Famennian (Fig 1).

#### Additional material

AM5242, MD plate (previously attributed to *A*. *doryssa* [2]); AM5922, MD plate with poorly preserved associated lateral trunk plate

#### Diagnosis

A small *Africanaspis* arthrodire with a high and broad median dorsal plate (L:H 2:3, with height measured obliquely along the front edge of the MD plate *sensu* [36]). Medium dorsal plate with posteriorly directed apex ornament comprising course dorsoventrally directed noded ridges and closely spaced, caudally directed tubercles along caudal margin.

#### Remarks

Long et al. [2] noted that one *Africanaspis* median dorsal plate (AM 5242) had a distinctly different ornament and height to length ratio compared with other assigned median dorsal plates and suggested that more than one species could be present. However, it was rather concluded that this variation was commensurate with ontogenetic variation and due to small sample size it was decided not to place this one specimen within a new species [2]. Based on new material, including adults and juveniles of *A*. *doryssa* and *A*. *edmountaini*, we can now determine that AM 5242 does in fact represent a separate species, being conspecific with *A*. *edmountaini*.

### Description

#### Dermal bones of the trunkshield

**Fig 12:**
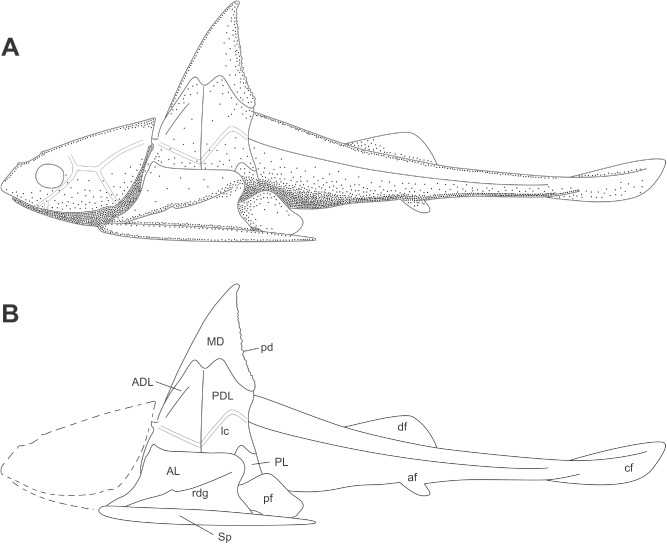
Whole bodied reconstruction *Africanaspis edmountaini sp*. *nov*. A, lateral view based on described material with the form of pectoral and caudal fins hypothetical, and head outline based on that of *A*. *doryssa* (above); B, outline drawing of A (not to scale).

As with *A*. *doryssa* the dorsal and lateral trunk shield plates are best preserved, with the median dorsal plate being the most common. The median dorsal plate is high and broad (L/H 2:<3) with caudally directed short serrations (Figs 9A; 10A and 10B, 10E; 11A and 12). It is ornamented with coarse noded vertical ridges (Figs 10A and 11A). The suture between the median dorsal plate and dorsal margins of the anterior dorsal and posterior dorsal plates is “m” shaped, the posterior arch on the “m” being higher than the anterior arch (Figs 10E; 11 and 12). A ventrally directed anterior process of the medium dorsal plate overlaps the anterior margin of the anterior dorsolateral plate, which slopes at a dorsocaudal angle of 40°. The ventral margin of the anterior dorsolateral plate has a concave curvature (Figs 9A and 10A). The posterior dorsolateral plate has a gently recurved concave caudal margin with a distinct caudally directed lobe immediately below the overlapping posterior margin of the median dorsal (Figs 9A and 10A). A triangular plate superimposed on the posteroventral margin of the posterior lateral plate in AM5921 is tentatively identified as a posterior lateral plate (Fig 10A and 10B).

The overall shape of the anterior lateral plate is rectangular, and the plate is longer than high (L/H+/- 3:2) (Figs 9B and 10C–10E). The ventrolateral margin of the anterior lateral plate is straight where it overlaps the spinal plate (Fig 9B). The anterior lateral’s anterior margin curves sharply upward and inward before reaching a point of dorsolateral inflexion whereafter the plate is more vertically directed and the anterior margin assumes a steeper lateral profile The dorsolateral inflexion of the anterior lateral plate contributes to the squat bell shape of the body in cross section. The spinal plate is incomplete, with only the mid medial and lateral margins preserved. There is an ornament of tubercles visible along the outer lateral margin similar to those in *A*. *doryssa*.

The outline of the mid body region and tail are preserved as an impression in AM5921a and b (Figs 9, 10F and 10G). A single, low dorsal fin is situated above the anal region of the trunk, and the anterior edge of the anal fin projects from the ventral margin of the body at the midline level of the dorsal fin (Figs 9A, 9B, 10F, 10G and 12). The body is twisted so that the tail is preserved in lateral view and the abdominal region is in ventral view. Within the abdominal cavity there is a carbonaceous mass, possible the intestine, visible inside the body wall, which has ruptured (Fig 10F–10G). The small anal fin has a wide base and a slightly narrower and rounded distal margin. The tail is long and tapers caudally although no part of the caudal fin is preserved. There are no ossified elements of the fin skeleton or axial skeleton preserved, however, the acidic nature of the host sediments may have resulted in the preferential dissolution of the perichondral bone [21].

### Reconstruction and comparison of *Africanaspis edmountaini* (Fig 12)

Currently there are no plates known from the head shield of *A*. *edmountaini* sp. *nov*. and the outline of the head of *A*. *doryssa* has been used to guide the reconstruction. The median dorsal plate is shorter and laterally wider than in *A*. *doryssa*. It has a far more robust vertical linear ornament than that of *A*. *doryssa* and the serration-like row of denticles along the posterior margin are shorter and caudally directed, as opposed to those of *A*. *doryssa* which are more elongated and more vertically directed. The anterior process on the median dorsal plate is thinner and does not extend as far towards the glenoid process as in *A*. *doryssa*, though as in *A*. *doryssa* the anterior margins of the median dorsal plate and anterior dorsolateral plate form a straight profile. The anterior lateral plate is not as deep as that of *A*. *doryssa*. The post thoracic region of the body is long and the dorsal fin is positioned far back along the dorsal margin of the tail. The trunk region immediately posterior to the thoracic armour is reconstructed as it is ruptured, probably due to the expulsions of gases of decomposition, and the specimen has probably suffered some post-mortem shrinkage of the soft tissues c.f. [45] such that the preserved margins of the tail are too narrow to conform to the caudal margin of the trunk shield.

## Soft tissue replacement and impressions in *Africanaspis*

In sites with exceptional soft tissue preservation, muscle is frequently preserved through authigenic or bacterially mediated phosphatization [32, 46]. The outer surface of the eye in the juvenile *A*. *doryssa* specimen (AM 5905 Fig 5A) has been replaced mainly by mica and possibly chlorite [21] and appears white, whereas the inner portion comprises black organic carbon. The mineral replacement has maintained the 3 dimensionality of the eyeball and it fills the orbital space, bulging outwards. Mineralized branchial, thoracic and trunk musculature has been previously reported in coccosteomorph arthrodires from the Gogo Formation, Western Australia; however, here the mineral of replacement is apatite [34, 47]. The paired otic capsules in both adult and juvenile specimens of *A*. *doryssa* also show 3D preservation. Decay sequence studies show the otic capsules to be more decay resistant than most other types of head tissues [33]. Within sharks the otic capsule remains articulated with the vertebral column up to the latter stages of the decay process [33] whereas other tissues of the head are reported to decay earlier [33].

In contrast to the poor preservation of the mineralised dermal skeleton of the Waterloo Farm fossils the body and tail are well preserved as 2D carbonaceous films, although as noted above there is no preservation of the axial skeleton (Figs 6; 8; 9; 10F and 10G). Similar styled preservation of body outlines, without mineralized myomeres, has been reported for *Cornovichthys blaauweni* [48], a jawless fish from Achanarras, Scotland and, *Austroptyctodus* and *Compagopiscis* from the Gogo Formation, Western Australia [34, 47]. In *Africanaspis* the outline of the anal and dorsal fins is preserved but not the outline of the caudal fin. In *Amphioxus* the smaller lobe of the caudal fin is the first fin to loose its shape during the decay processes [33] and it appears that the same sequence of decay occurred in *Africanaspis*. The dorsal fin in *Africanaspis* is preserved well back on the body, level with the position of the anal fin. In modern lampreys and sharks the first smaller dorsal fin decays prior to the second larger dorsal fin [33] and so the lack of preservation of an anterior dorsal fin does not prove that *Africanaspis* lacked a more anterior, first dorsal fin. However, within placoderms a single median dorsal fin (as indicated by fin radials) commonly occurs posterior to the pelvic fins, above the claspers in arthrodires and more posteriorly in ptyctodonts [49] and antiarchs, where it is preserved as an impression, without fin radials, in both *Parayunnanolepis xitunensis* [50] and *Bothriolepis canadensis* [51].

The dorsal margin of the body appears to be intact, although the ventral body margin is not completely preserved and has ruptured to reveal a tubed shape containing a mass of dark carbonaceous matter, possibly the gut, in AM 135921 (Fig 10F and 10G). In other placoderms *Bothriolepis* [52] and in acanthodians [53], which show similar preservation to the africanaspids, comparable structures have been interpreted as the gut.

## Discussion

### Ecology

The sheltered estuarine depositional environment, in which the fossils have been recovered, was first suggested as a nursery site due to the abundance of antiarch and arthrodire juveniles [3]. Further work described shark [7] and coelacanth embryos [8] indicating the use of this environment as a nursery by multiple taxa. Coelacanths were almost exclusively represented by small juveniles within a narrow size range, suggesting use of the environment as a nursery by adults from an adjacent environment [8]. Conversely arthrodires are represented by a full range of sizes suggesting use of quieter parts of the environment as nurseries by adults also inhabiting the estuary. Within modern ecosystems a nursery is generally defined as an area in which juveniles occur in higher densities than adults, and/or obtain a greater growth rate than they are able to in other habitats [54]. Although comparison of growth rate between different habitats was precluded in this study, the presence of a greater number of juveniles than adults of *Africanaspis*, conforms to this definition. Studies on extant show that few species are limited to a single nursery habitat, the majority utilizing a mosaic of connected habitats due to tidal conditions, feeding, protection and cover seeking behaviours [55–58]. The difficulty in incorporating dynamic processes such as ontogenetic niche shift and habitat connectivity within the definition of a nursery site is recognized, with most approaches criticized for being static [54]. The very nature of the fossil record compounds these problems when defining paleonurseries. *Contra* [59] sites with a large number of juveniles preserved such as Lode Quarry are here considered as nursery sites in accordance with the definition of multi species nurseries which recognize multiple contributing factors of habitat and connectivity [60, 61]. In sites such as the Gogo Formation, where intrauterine embryos [14, 17, 49, 62, 63] and ontogenetic series are preserved for many of the arthrodire taxa [12, 64–66], the numbers of juvenile individuals are notably less than adults and so therefore this does not represent a nursery.

Despite the preservation of embryos the reproductive strategy of *Africanaspis* remains unknown. The small size of AM7503, identified as a neonate, suggests hatching or birthing within the estuarine environment. However, although juveniles dominate this site, the numbers are not indicative of a spawning site such as Lode Quarry, Latvia or Tioga County, Pennsylvania. Internal fertilization with egg laying (oviparity) is a conceivable strategy. However, internal fertilization with live birth (viviparity) cannot be ruled out as a mode of reproduction because embryos recovered from within specimens of *Incisoscutum* [14] fall within the size range of the smallest individuals of *Africanaspis* known. Although internal fertilization, with either oviparity or viviparity correspond with the number and size of embryos recovered for *Africanaspis*, to date no evidence of sexual dimorphism, an indicator of internal fertilization within vertebrates, is known for *Africanaspis*. It is however pertinent that the presence of intromittant organs is not a prerequisite for internal fertilisation. Modern coelacanths lack such organs but bear live young [67, 68], as did ancient coelacanths such as Jurassic *Undina pencillata*, [69]

Olive et al. [70] have drawn attention to faunal and floral similarities between the paleogeographically adjacent Famennian sites of the Catskill Formation, Red Hill Pennsylvania [36], and Strud in Belgium [70]. Notably there are also strong similarities between these two sites and Waterloo Farm. Red Hill and Waterloo Farm uniquely share the presence of a *Hyneria*-like tristichopterid sarcopterygian, in addition to gyracanthid acanthodians, actinopterygians, a dipnoan, a species of *Groenlandaspis*, an *Africanaspis*-like arthrodire [71, 72], and undescribed sarcopterygii [per. obs]. Strud and Waterloo Farm likewise share an *Africanaspis*-like arthrodire, actinopterygians, dipnoans and a tristichopterid sarcopterygian. In addition they both have acanthodii and a bothriolepid antiarch [68, [72]. The main differences are the lack of phyllolepids from Waterloo Farm, together with the striking taxonomic differences in the chondrichthyan faunas of the Red Hill and Waterloo sites and the lack of chondrichthyans from the Strud locality. The Strud and Red Hill fossil sites are both interpreted as fresh water tropical Laurasian deposits whereas Waterloo Farm is interpreted as a high paleolatitude Gondwanan estuarine environment, with significant fresh water input [10]. It is possible that slight high latitude climatic amelioration towards the end of the Devonian [73], coupled with increasing proximity of Laurasia and Gondwana [74] permitted a range of coastal and lowland aquatic vertebrate taxa to extend over a wide geographic range. The presence of multiple juveniles representing different species [3,7,8] from the Waterloo Farm fossil site indicates the ability of these taxa to survive and reproduce at high latitudes.

### Taxonomy

We agree with Daeschler et al. [34] that it would be premature to resurrect the tiaraspididae of Miles [24], but like Daeschler we note strong similarities between *Turrisaspis*, *Tiaraspis* and *Africanaspis*. Notably they share a distinctive high-spired median dorsal plate (H:L index >1) separating the ADL plates and high cranio-caudally shortened dorsolateral trunk armour. They share with some but not all species of *Groenlandaspis*, a raised crest running from the condyle of the ADL plate to centre of the caudal margin thereof and a nuchal plate lacking a median boss (though sometimes exhibiting a small posterior median crest). Though unconfirmed in *Tiaraspis* they most likely also share the presence of two pineal plates. A close relationship between *Africanaspis*, *Turrisaspis* and *Tiaraspis* was previously questioned [36] on the basis of the ADL and PDL plates in *Africanaspis*, as described by Long et al. [2], not being cranio-caudally shortened to the same extent as seen in the other two genera. Our new material indicates that the trunk armour was indeed shortened cranio-caudally and is intermediate in length between those of *Tiaraspis* and *Turrisaspis*. In addition we note that this study further demonstrates that contra Long *et al* [2] and Olive *et al*[59] the MD plate of *Africanaspis doryssa* does not become broader in lateral view with age, as this interpretation resulted from confusion of two discrete species. Publication, for the first time of *Africanaspis* head material reveals further similarities between *Africanaspis* and *Turrisaspis*.

## Conclusion

Waterloo Farm has been interpreted as a high latitude estuarine environment and to date 370 identifiable fish fossils (excluding dissociated fish scales) have been recovered. The dominant preserved component of the fauna were placoderms (58%) and of these 76% were arthrodires, however, only 8% of arthrodire specimens have been identified as belonging to the genus *Africanaspis*, making it a rare component of the placoderm fauna. Newly prepared and collected material including one specimen with associated head and trunk shield plates (AM4907), an additional adult skull (AM 5943) and the complete armour of a juvenile (AM 5905) has resulted in a headshield originally attributed as a juvenile of *Groenlandaspis riniensis* [2] being reassigned to *A*. *doryssa* (AM 4907). In addition, impression of soft tissue morphology has allowed parts of the post thoracic body and fins to be described within the genus *Africanaspis* for the first time. Variation previously attributed to ontogeny within *A*. *doryssa* is diagnostic for a new species *A*. *edmountaini*. Full size ranges of specimens, from neonatal to presumed adults, suggest that *Africanaspis* was a resident reproducing within the estuarine setting of deposition. Intriguing similarities and differences are noted between the faunas of the *Africanaspis*-bearing Waterloo Farm, and *Turrisaspis*-bearing Red Hill, Pennsylvania and Strud Quarry, Belgium sites. The high paleolatitude and Gondwanan setting of Waterloo Farm contrasts with the equatorial paleolatitude and Laurasian setting of the latter sites. Marked similarities between them support possible reduction in faunal provincialism towards the end of the Devonian [74].
